# Macular Retinal Vessel Oxygen Saturation Elevation in Chinese Central Serous Chorioretinopathy

**DOI:** 10.1155/2017/5972418

**Published:** 2017-11-02

**Authors:** Cheng Li, Xiaoxiao Feng, Xin Wen, Xiaobing Qian, Qishan Zheng, Yongguang Yuan, Yue Fu, Wanwen Shao, Yujie Li, Xiaofeng Lin, Qianying Gao

**Affiliations:** ^1^State Key Laboratory of Ophthalmology, Zhongshan Ophthalmic Center, Sun Yat-sen University, Guangzhou 510060, China; ^2^Sun Yat-sen Memorial Hospital, Sun Yat-sen University, Guangzhou 510060, China

## Abstract

**Purpose:**

To evaluate the retinal vessel oxygen saturation in central serous chorioretinopathy (CSC) cases among the Chinese.

**Methods:**

Relative oxygen saturation of retinal blood vessels was measured in 33 Chinese patients with single-eye CSC using the Oxymap T1 retinal oximeter. The contralateral eyes were the control. The mean saturation of the retinal arteriole (AS) and venule (VS), arteriovenous difference (AVS), and arteriole and venule diameters (AD, VD) was analyzed in the optic disc area and macular region.

**Results:**

In the optic disc area, the inferotemporal quadrant (TI) AS (93.2 ± 10.2%) and inferonasal quadrant (NI) VS (61.3 ± 7.3%) were higher in the affected eyes than in the contralateral eyes (88.7 ± 7.7% and 56.9 ± 6.5%) and AVS in NI (36.7 ± 10.4%) decreased compared to the contralateral eyes (41.5 ± 11.2%). The VD in TI was expanded (19.9 ± 2.5 pixels versus 18.1 ± 3.4 pixels). Around the macular region, AS was 93.6 ± 7.6%, higher than in the contralateral eyes (89.5 ± 6.3%). No other significant changes were found.

**Conclusions:**

AS increased in the TI, and VS decreased in the NI in the eyes with CSC. In addition, AS also increased around the macular region, suggesting that these are contributors to CSC pathophysiology.

## 1. Introduction

Central serous chorioretinopathy (CSC) is a posterior segment disease characterized by the serous detachment of the neurosensory retina in the macular area [1]. CSC occurs in 9.9 per 100,000 men and 1.7 per 100,000 women, six times more frequently in men than in women [2]. This disease can be classified as acute (less than three to six months in duration) or chronic (longer term) [3]. As a benign and self-limiting disease, most acute CSC cases last only between two and three months [4]; however, CSC recurs in approximately one-third of recovered patients [5]. The symptoms of acute CSC are related to the localization of the subretinal detachment around the macular area and limited focal or multifocal retinal pigment epithelium (RPE) alterations. Chronic CSC may also cause severe loss of visual acuity and decreased light sensitivity [6].

Advances in imaging techniques, such as fluorescein fundus angiography (FFA) and indocyanine green angiography (ICGA), have enabled researchers to obtain a better understanding of the anatomical structural alterations occurring in CSC. They include disturbed choroidal circulation and retinal pigment epithelium (RPE) which are the primary changes associated with this disease. The current understanding of the pathophysiology of CSC involves choroidal vascular hyperpermeability, which leads to increased tissue hydrostatic pressure beneath the RPE and discontinuity of the RPE [7]. ICGA findings showed staining of the inner choroid in the midphase of the angiogram, suggesting that choroidal vascular hyperpermeability may arise from venous congestion and ischemia [8]. Some studies found that patients affected by CSC often have higher levels of serum and urinary cortisol and catecholamines than do healthy subjects and therapies with local or systemic steroids can cause the disease [9–11]. Glucocorticoid elevations were also identified as the primary risk factor for the onset of CSC [12–14]. However, some regions of the retina may show signs of increased choroidal hyperpermeability but are not linked to manifestations of CSC [8]. Additionally, Turkcu et al. found that the antioxidant capacity decreased significantly in CSC cases, which implies that the oxidative process is involved in the pathogenesis of CSC [15].

Because the choroidal vessels contribute greatly to CSC pathophysiology and the ophthalmic artery supplies the retinal circulation, this study investigated whether altered retinal circulation was also related to CSC. Noninvasive spectrophotometric retinal oximetry has been used to measure oxygen saturation in retinal arterioles and venules [16]. This method was applied to determine whether there is an association between changes in retinal vessel oxygen saturation and the vessel diameter around the macular region in patients with CSC. In this study, we used a noninvasive retinal oximeter to measure oxygen saturation in retinal vessels in patients with CSC to detect retinal oxygen metabolic changes.

## 2. Materials and Methods

The study protocol was reviewed and approved by the Sun Yat-sen University Medical Ethics Committee (Zhongshan Ophthalmic Center Medical Ethics (2013) number 07). The protocol strictly adhered to the principles of the World Medical Association's Declaration of Helsinki. Informed consent was obtained from all subjects before examinations were performed.

### 2.1. Subjects

The study included the following: 33 CSC patients, of which 20 patients were diagnosed with acute CS and 13 patients were diagnosed with chronic CSC. The inclusion criteria for the CSC patients were the following: Chinese (xanthoderm origin), one eye featuring typical funduscopic appearance for CSC, normal binocular intraocular pressure (10–21 mmHg), and no other ocular disease or history of eye surgery. The manifestations of CSC include serous retinal detachment as well as RPE detachment or dysfunction without evidence of any other possible cause of exudation, such as inflammation, infiltration, or choroidal neovascularization. The eligible patients were those in whom only one eye exhibited manifestations of CSC, and their contralateral eyes were measured as the control.

Exclusion criteria were as follows: congestive heart failure, diabetes mellitus, coronary artery disease, uncontrolled arterial hypertension, hyperlipidemia, tumors, autoimmune and inflammatory diseases, renal and hepatic disorders, endocrine pathology, and concomitant treatment affecting androgen metabolism (statins and calcium antagonists) as well as drug and/or alcohol abuse. Additionally, subjects with ametropia of greater than 3D or anisometropia of less than 1D were excluded.

All patients were diagnosed by the same ophthalmologist and diagnoses were verified according to the images from fluorescein angiography and optical coherence tomography (OCT). Additionally, all participants underwent complete ophthalmic examinations consisting of the best-corrected visual acuity (LogMAR visual acuity chart), refraction, and intraocular pressure (IOP) examined using a noncontact tonometer (NCT) (Canon TX-20), a slit-lamp examination (Suzhou YZ5S), fundoscopy (Topcon TRC-50DX), and OCT (Topcon 3D OCT-1000). Finger pulse oximetry (Biolight M70), blood pressure, and heart rate (BangPu BF-1100) were measured before retinal oximetry. The ocular perfusion pressure (OPP) was calculated using the following equation:

(1) $$\begin{array}{c} OPP=\left(\frac{2}{3}\right)\left(\left(\frac{2}{3}\right){BP}_{diast}+\left(\frac{1}{3}\right){BP}_{syst}\right)-IOP, \end{array}$$

where BP_diast_ and BP_syst_ are the diastolic blood pressure and systolic blood pressure, respectively [17].

Complete medical histories were obtained for all test patients, including hormonal abnormalities, renal disease, hypertension, smoking, and medications that may alter hormonal status, such as glucocorticoids, statins, blockers, psychotropics, hormonal replacement therapy, or hormonal inhibitors.

### 2.2. Retinal Oximetry

The retinal oximeter used in this study (Oxymap ehf., Reykjavik, Iceland) has been described in detail previously [18]. It features a fundus camera base coupled with a beam splitter and a digital camera, yielding fundus images at two light wavelengths simultaneously. Specialized software automatically selects the measurement points on the oximetry images and calculates the optical density (absorbance) of the retinal vessels at two wavelengths, 600 nm and 570 nm. The optical density is sensitive to oxygen saturation at 600 nm; however, the optical density is not sensitive to oxygen saturation at the reference wavelength of 570 nm. The ratio of these optical densities is approximately linearly related to the hemoglobin oxygen saturation. The oximeter was calibrated to yield the relative oxygen saturation values. The calibration is not perfect, however, and in some cases, the measurement exceeds 100%. The oximeter is sensitive to changes in oxygen saturation and to yield repeatable results. The oximetry values obtained may thus be used for comparison, although they may differ from the absolute saturation values.

The pupils of each subject were dilated with 0.5% tropicamide (Shenyang Xingji Co., Shenyang, China) for at least 15 minutes before retinal oximetry measurements or until the pupils were dilated to 5.5 to 6.0 mm in diameter. The same skilled photographer took images in a dark room; light was provided only from the fundus camera and the computer screen. The standard procedure and parameters were the following: (1) lowest illumination intensity, (2) flash intensity = 50 W·s [19], (3) small aperture and small pupil, (4) consistent angle of gaze with optic disc located at the image center, and (5) consistent order of photographs (right eye first, three images for each eye). The time between the images was approximately 1 minute, and the best quality image with the optic disc in the center was selected for analysis.

### 2.3. Image Analysis

**Figure 1:**
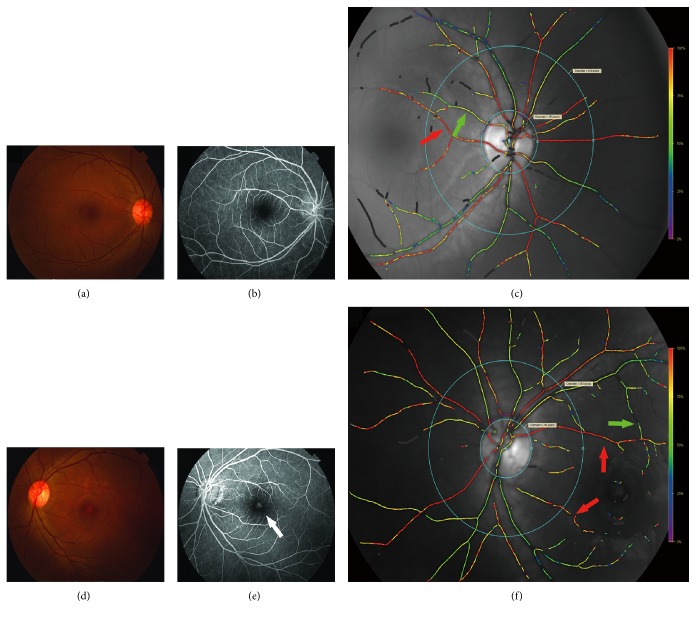
Fundus photograph, FFA, and the pseudocolor fundus image. (a), (b), and (c) are images of the contralateral eye of one patient, while (d), (e), and (f) are images of the CSC-affected eye from the same patient. (a) and (d) are fundus photograph images. (b) and (e) are fundus fluorescein angiography (FFA) images. The white arrow shows the leakage in the macular region of the CSC-affected eye. (c) and (f) are the pseudocolor fundus images. Vessels of four quadrants were selected between the two circles, and the red arrows show the arteries selected for measuring AS; similarly, the green arrows show the venules selected for measuring VS.

The oxygen saturation and the width of the retinal vessels were analyzed automatically using the Oxymap Analyser version 2.0 specialized software, and at the same time, it measured the fundus image quality, which takes into account overall quality, focus, and contrast. The analyzer treated oximetry images from the affected eyes and contralateral eyes equally. The pseudocolor fundus image provides a quick overview of the saturation distribution in the retinal vessels (Figure 1). The retinal oxygen levels of the optic disc area and macular region (the arterioles and the venules) were measured separately.

The vessel selection method was the following. Using the center of the optic disc as the origin, one small circle just on the rim of the optic disc and another circle three times larger were drawn on the pseudocolor fundus images of all patients. The vessels between the two circles were selected to be analyzed overall and in four quadrants, that is, the superonasal quadrant (NS), the inferonasal quadrant (NI), the inferotemporal quadrant (TI), and the superotemporal quadrant (TS). This is the method that Geirsdottir et al. used [20] to measure the oxygen saturation in the optic disc area. Then, the arterioles and the venules between the superior and the inferior vessel arches were analyzed. The first and second branches of the vessels with widths of eight or more pixels were selected for analysis. The images and selection methods are shown in Figure 1.

### 2.4. Statistical Analyses

Statistical analyses were performed using the program SPSS 10.0 for Windows. Descriptive statistics were calculated for clinical data. For the statistical analysis between affected eyes and contralateral eyes, the paired *t*-test or Wilcoxon matched pair signed rank sum test was employed to evaluate group difference significance between different quadrants according to their distribution test results (Shapiro–Wilk test; *α* = 0.05): paired *t*-test for normality, Wilcoxon matched pair signed rank sum test for abnormal distribution. The data are expressed as mean ± standard deviation. All statistical tests were two sided, and *p* < 0.05 was considered statistically significant. For the statistical analysis between acute and chronic patients, the statistic method used was the unpaired *t*-test or rank sum test (Mann–Whitney *U* test) depending on the distribution of the data (Shapiro–Wilk test; *α* = 0.05) and homogeneity of variance (*F* test; *α* = 0.05): the unpaired t-test was used for normal distribution and homogeneous variance, while the rank sum test (Mann–Whitney *U* test) was used for abnormal distribution or inhomogeneous variance.

## 3. Results

A total of 33 single eye-affected patients were recruited for the retinal vessel oxygen measurements with the contralateral eyes serving as the control. The systemic conditions and eye measurement data are shown in Table 1.

Because CSC is six times more common in men than in women [2], most of the clinic patients were men; only five women were included in this study. This difference in prevalence is consistent with that in other studies.

**Figure 2:**
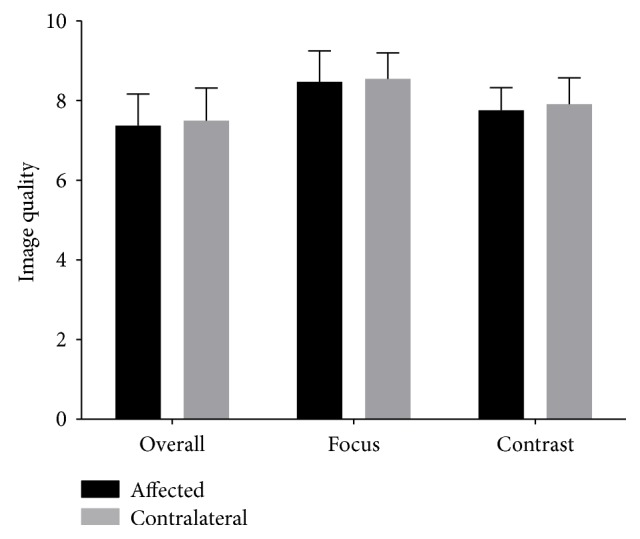
Image quality scores between the affected eyes and contralateral eyes. No difference was significant between the affected and contralateral eyes in overall quality, focus, or contrast scores. The paired *t*-test was used in the overall and focus comparison, resulting in *p* values of 0.194 and 0.400, respectively. The Wilcoxon matched pair signed rank sum test was used in the contrast comparison, resulting in a *p* value of 0.129.

The Oxymap Analyser software generated three image quality scores for each image based on overall quality, focus, and contrast. Out of these images, only one patient's fundus image quality assessment was less than 6. In his affected eye, overall assessment was 5 and focus assessment was 5.5, while in the contralateral eye, overall assessment was 5.1. Most of the patients scored more than 7, and five patients scored between 6 and 7. The difference in image quality between affected eyes and contralateral eyes was not significant; details are shown in Figure 2 (overall: paired *t*-test, *p* value was 0.195; focus: paired *t*-test, *p* value is 0.400; contrast: Wilcoxon matched pair signed rank sum test, *p* value is 0.129).

First, we used Shapiro–Wilk test to assess the distribution of oxygen saturation and the changes in vessel diameters between affected eyes and contralateral eyes. Only the overall AS, NI AS, overall VS, and TS AVS were not normally distributed, and these were analyzed via the Wilcoxon matched pair signed rank sum test. The numerical values and statistical results for the overall optic disc area and four quadrants are shown in Table 2.

Remarkably, the AS in the TI increased (93.2% ± 10.2%) compared to that in the contralateral eyes (88.7% ± 7.7%). In the NI of the affected eyes, the VS increased compared to that in the contralateral eyes, 61.3% ± 7.3% versus 56.9% ± 6.5%, respectively. On the contrary, in the NI, the AVS decreased (36.7% ± 10.4%) compared to that in the contralateral eyes (41.5% ± 11.2%), which means CSC causes oxygen consumption to rise. Additionally, VD in TI increased from 18.1 ± 3.4 pixels in the contralateral eyes to 19.9 ± 2.5 pixels in the affected eyes.

**Figure 3:**
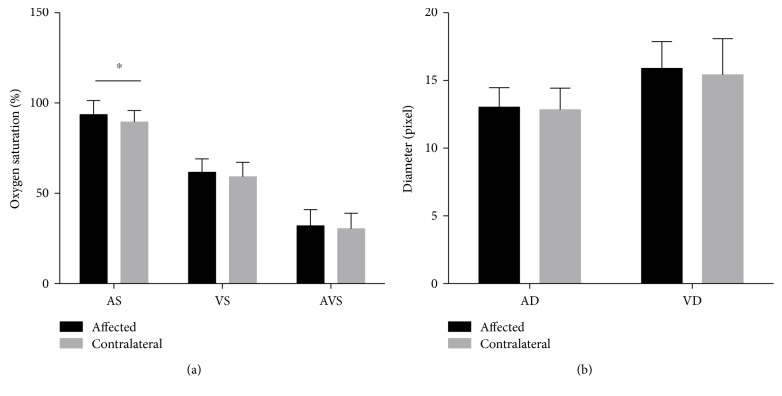
Oxygen saturation and vessel diameters around the macular area. (a) Compared to the contralateral eyes, AS is increased in the affected eyes (*p* value of 0.006), but AVS shows no significant difference. ^∗^*p* < 0.05 (AS: Wilcoxon matched pair signed rank sum test; VS, AVS: paired *t*-test). (b) Vessel diameters show no change. ^∗^*p* < 0.05 (paired *t*-test).

Because CSC primarily influences the macular area lying on the temporal side [8], the rise in oxygen saturation may be a result of alterations between macular area oxygen replenishment and consumption. To test this possibility, we also selected arterioles and venules between the superior and inferior vessel arches to analyze the oxygen saturation around the macular area. These results are shown in Figure 3. The arteriole oxygen saturation in the affected eyes is 93.6% ± 7.6%, while in the contralateral eyes, it is only 89.5% ± 6.3%, and the *p* value is 0.006 (Wilcoxon matched pair signed rank sum test). However, the AVS showed no significant change.

Then, we compared the acute and chronic CSC patients to determine whether the CSC type influenced the results. We compared the affected eyes between acute and chronic patients (Table 3) and also compared the contralateral eyes (Table 4) between acute and chronic patients. The areas compared are the overall area, the four quadrants of the optic disc, and the macular region. There was no significant difference in the oxygen saturation or vessel diameter between acute and chronic CSC patients overall, in the four optic disc quadrants, or in the macular region. And it also proved that it was reasonable to combine these data for analysis.

Additionally, using data from a previously published paper [18] from our group that considered 129 local healthy young people's oxygen and diameter data, we reanalyzed our data against data from patients in which neither eye shows CSC, and we found that in the CSC-affected eyes, AS obviously increased in the NS, TI, and TS quadrants, excepting only the NI quadrant, and the VS decreased in TI. As for diameter, the venules expanded in the NS, TI, and TS quadrants. At the same time, in the contralateral eyes, AS was higher in TS, and the VS was also lower than in healthy eyes in N and TI even though patients were not matched for age and gender. These data are shown in Supplementary Tables 1 available online at https://doi.org/10.1155/2017/5972418 and 2.

## 4. Discussion

We show here that retinal vessel saturation may change in CSC-affected eyes. When these levels in CSC-affected eyes were compared to those in the contralateral eyes of the same patient, changes were found in the TI AS and the NI VS, and the AS was moderately increased around the macular region. Additionally, the contralateral eyes had higher mean AS and lower mean VS in specific quadrants when compared to the eyes of healthy young Chinese from our previous study, despite not being matched for age and gender [18]. This implies that CSC, a systemic disease, may also have effects on the contralateral eyes, rendering them unhealthy. This complication may in turn hinder assessment of the actual changes caused by CSC.

Researchers have reported that during the process of CSC, the choroidal blood flow decreases and the subfoveal choroidal blood flow is abnormally regulated [21]. Therefore, if the change in retinal vessel oxygen saturation around the macular region in CSC is a result of a change in the retinal vessel blood flow, then such effects still need to be more rigorously evaluated. Moreover, it has been shown that the oxygen saturation levels changed in some other eye diseases as well. For example, AS was significantly higher in patients with retinitis pigmentosa (RP) than in normal subjects among 20- to 40-year-old subjects and in elderly subjects. However, AS was significantly lower in RP patients than it was in healthy subjects [17]. In patients with an ischemic branch due to retinal vein occlusion (BRVO), the occluded arteriole oxygen saturation increased compared to the saturation levels in vessels from the same quadrant in the contralateral eyes [22]. Our group also found decreased retinal arteriole saturation, a decreased difference in arteriovenous saturation, and a narrowing of retinal vessel diameter in highly myopic eyes [23]. These data show that changes in oxygen saturation levels are an essential component of eye diseases.

However, this pilot clinical study has some limitations. The changes that occurred in the eyes of CSC patients must be further verified. The changes in this study, while significant, are small and only exist in the quadrants inclusive of the macular area. The reliability of these measurements warrants future validation. Additionally, CSC is a disease related to systemic conditions, which makes it necessary to increase the sample size and add normal eyes from age- and gender-matched persons as controls in additional studies. The oximetry analysis of oxygen saturation is based on the absorption of two wavelengths of light. Such dependence can be problematic if changes occur in the fundus pigment or if cataracts are present. Specifically, changes in the blood component profiles during CSC can cause measurement errors, which may induce a false-positive conclusion when analyzing the AS around the macular region. Another limitation is that we could not perform consecutive oxygen saturation measurements during the disease process for comparison with data collected after recovery.

However, considering that there is currently no consensus on the optimal method of measuring oxygen saturation in the retinal vasculature, oximetry is still a relatively safe and effective way to obtain such measurements. In summary, the results provide valuable insight that will help to guide further research on CSC.

## 5. Conclusions

This study shows that AS increased during the CSC process in TI regions within the optic disc area and around the macular region; in addition, VS decreased in NI, which provides data requiring further study to confirm that oxygen saturation changes play an important role in the CSC process.

## Supplementary Material

Table 1. Comparison of oxygen saturation and vessel diameter between CSC affected eyes and healthy young people's normal eyes. Table 2. Comparison of oxygen saturation and vessel diameter between CSC patients' contralateral eyes and healthy young people's normal eyes.

## Figures and Tables

**Table 1:** Characteristics of the study subjects.

Variables	Systemic conditions	Contralateral eyes	Affected eyes
Gender (male) *N* (%)	28 (85%)	——	——
Gender (female) *N* (%)	5 (15%)	——	——
Age (year)	41 ± 8	——	——
Systolic blood pressure (mmHg)	119 ± 16	——	——
Diastolic blood pressure (mmHg)	80 ± 13	——	——
Pulse	79 ± 14	——	——
Finger oximetry (%)	98 ± 1	——	——
Visual acuity	——	1.0 ± 0.4	0.5 ± 0.3
Intraocular pressure (mmHg)	——	15 ± 3	15 ± 3
Perfusion pressure (mmHg)	——	55 ± 9	55 ± 9

**Table 2:** Comparison between affected eyes and contralateral eyes.

	Variables	Affected eyes	Contralateral eyes	Statistical method	*p* value
AS (%)	Overall	96.6 ± 7.0	95.4 ± 6.7	Wilcoxon test	0.623
	NS	101.2 ± 7.9	98.9 ± 8.1	Paired *t*-test	0.054
	NI	98.0 ± 10.2	98.5 ± 11.6	Wilcoxon test	0.993
	TI	93.2 ± 10.2	88.7 ± 7.7	Paired *t*-test	0.049^∗^
	TS	94.5 ± 8.0	93.1 ± 6.2	Paired *t*-test	0.238

VS (%)	Overall	60.9 ± 6.2	58.8 ± 6.3	Wilcoxon test	0.053
	NS	62.4 ± 7.1	61.0 ± 7.6	Paired *t*-test	0.286
	NI	61.3 ± 7.3	56.9 ± 6.5	Paired *t*-test	0.005^∗^
	TI	53.4 ± 8.7	52.8 ± 9.0	Paired *t*-test	0.761
	TS	61.1 ± 8.8	61.3 ± 8.6	Paired *t*-test	0.874

AVS (%)	Overall	35.7 ± 6.5	36.6 ± 6.3	Paired *t*-test	0.508
	NS	38.8 ± 9.3	37.9 ± 9.7	Paired *t*-test	0.489
	NI	36.7 ± 10.4	41.5 ± 11.2	Paired *t*-test	0.024^∗^
	TI	39.8 ± 10.7	35.9 ± 9.9	Paired *t*-test	0.188
	TS	33.4 ± 10.7	31.7 ± 8.5	Wilcoxon test	0.458

AD (pixel)	Overall	13.0 ± 1.2	13.0 ± 1.2	Paired *t*-test	0.776
	NS	12.8 ± 1.8	13.0 ± 1.8	Paired *t*-test	0.683
	NI	12.5 ± 1.8	12.7 ± 1.7	Paired *t*-test	0.56
	TI	13.8 ± 2.5	13.8 ± 2.5	Paired *t*-test	0.88
	TS	13.4 ± 2.0	14.2 ± 2.0	Paired *t*-test	0.071

VD (pixel)	Overall	17.3 ± 1.4	16.8 ± 1.7	Paired *t*-test	0.105
	NS	17.1 ± 2.1	16.8 ± 2.2	Paired *t*-test	0.381
	NI	15.8 ± 2.9	16.1 ± 2.8	Paired *t*-test	0.676
	TI	19.9 ± 2.5	18.1 ± 3.4	Paired *t*-test	0.01^∗^
	TS	18.1 ± 2.7	17.0 ± 2.8	Paired *t*-test	0.129

^∗^ *p* < 0.05.

**Table 3:** Comparison of oxygen saturation and vessel diameter of the affected eyes between acute and chronic CSC patients.

	Variables	Acute CSC	Chronic CSC	Statistical method	*p* value
AS (%)	Overall	97.1 ± 7.8	95.7 ± 5.8	Unpaired *t*-test	0.565
	NS	100.5 ± 7.3	102.3 ± 9.0	Unpaired *t*-test	0.526
	NI	97.5 ± 11.8	98.7 ± 7.3	Unpaired *t*-test	0.746
	TI	94.4 ± 10.4	91.3 ± 10.0	Mann–Whitney *U* test	0.461
	TS	94.8 ± 9.5	94.1 ± 5.2	Mann–Whitney *U* test	0.854
	Macular region	94.5 ± 8.0	92.3 ± 7.5	Unpaired *t*-test	0.444

VS (%)	Overall	59.9 ± 6.7	62.4 ± 5.1	Unpaired *t*-test	0.248
	NS	62.2 ± 7.6	62.6 ± 6.7	Unpaired *t*-test	0.884
	NI	60.9 ± 7.7	61.8 ± 7.0	Unpaired *t*-test	0.735
	TI	52.4 ± 10.5	54.9 ± 4.7	Mann–Whitney *U* test	0.507
	TS	61.2 ± 5.7	60.9 ± 12.5	Mann–Whitney *U* test	0.883
	Macular region	62.2 ± 8.5	60.8 ± 5.7	Unpaired *t*-test	0.609

AVS (%)	Overall	37.3 ± 6.4	33.2 ± 6.2	Unpaired *t*-test	0.083
	NS	38.2 ± 6.6	39.7 ± 12.6	Unpaired *t*-test	0.854
	NI	36.6 ± 9.3	36.9 ± 12.4	Mann–Whitney *U* test	0.938
	TI	42.0 ± 11.9	36.4 ± 7.8	Unpaired *t*-test	0.145
	TS	33.6 ± 8.8	33.2 ± 13.4	Mann–Whitney *U* test	0.658
	Macular region	32.3 ± 10.1	31.5 ± 7.1	Unpaired *t*-test	0.813

AD (pixel)	Overall	13.2 ± 1.3	12.6 ± 1.0	Unpaired *t*-test	0.107
	NS	12.8 ± 1.6	12.8 ± 2.2	Unpaired *t*-test	0.886
	NI	12.8 ± 1.9	12.0 ± 1.7	Unpaired *t*-test	0.233
	TI	14.1 ± 2.7	13.4 ± 2.1	Unpaired *t*-test	0.450
	TS	13.8 ± 2.2	12.7 ± 1.5	Unpaired *t*-test	0.116
	Macular region	13.2 ± 1.3	12.8 ± 1.6	Unpaired *t*-test	0.373

VD (pixel)	Overall	17.6 ± 1.5	16.8 ± 1.2	Unpaired *t*-test	0.152
	NS	17.1 ± 2.1	17.2 ± 2.2	Unpaired *t*-test	0.877
	NI	16.0 ± 3.0	15.6 ± 2.8	Unpaired *t*-test	0.731
	TI	20.3 ± 2.8	19.3 ± 2.1	Unpaired *t*-test	0.289
	TS	18.0 ± 2.4	18.1 ± 3.1	Unpaired *t*-test	0.874
	Macular region	15.8 ± 2.0	16.1 ± 1.9	Unpaired *t*-test	0.625

**Table 4:** Comparison of oxygen saturation and vessel diameter of the contralateral eyes between acute and chronic CSC patients.

	Variables	Acute CSC	Chronic CSC	Statistical method	*p* value
AS (%)	Overall	95.9 ± 6.6	94.6 ± 7.0	Unpaired *t*-test	0.591
	NS	98.4 ± 7.0	99.7 ± 9.7	Unpaired *t*-test	0.660
	NI	98.5 ± 11.3	98.5 ± 12.4	Unpaired *t*-test	0.992
	TI	88.4 ± 8.1	89.2 ± 7.5	Unpaired *t*-test	0.764
	TS	94.0 ± 6.6	91.7 ± 5.4	Unpaired *t*-test	0.300
	Macular region	90.1 ± 5.3	88.6 ± 8.0	Mann–Whitney *U* test	0.397

VS (%)	Overall	59.3 ± 7.2	58.2 ± 4.9	Unpaired *t*-test	0.645
	NS	62.0 ± 9.0	59.4 ± 4.5	Mann–Whitney *U* test	0.418
	NI	58.0 ± 6.8	55.4 ± 6.0	Mann–Whitney *U* test	0.269
	TI	52.8 ± 9.0	52.7 ± 9.4	Unpaired *t*-test	0.996
	TS	62.5 ± 9.1	59.5 ± 7.7	Mann–Whitney *U* test	0.357
	Macular region	60.2 ± 7.4	57.5 ± 8.9	Unpaired *t*-test	0.339

AVS (%)	Overall	36.7 ± 5.8	36.4 ± 7.3	Unpaired *t*-test	0.914
	NS	36.4 ± 8.7	40.2 ± 10.9	Unpaired *t*-test	0.269
	NI	40.5 ± 10.4	43.1 ± 12.5	Unpaired *t*-test	0.520
	TI	35.6 ± 9.0	36.5 ± 11.6	Unpaired *t*-test	0.812
	TS	31.5 ± 8.7	32.1 ± 8.6	Mann–Whitney *U* test	0.685
	Macular region	29.9 ± 8.4	31.2 ± 9.1	Unpaired *t*-test	0.685

AD (pixel)	Overall	13.0 ± 1.2	13.1 ± 1.4	Unpaired *t*-test	0.838
	NS	12.8 ± 1.7	13.3 ± 2.1	Unpaired *t*-test	0.385
	NI	12.9 ± 1.8	12.4 ± 1.5	Unpaired *t*-test	0.433
	TI	14.2 ± 2.5	13.1 ± 2.5	Mann–Whitney *U* test	0.185
	TS	13.9 ± 1.7	14.8 ± 2.4	Unpaired *t*-test	0.218
	Macular region	13.0 ± 1.5	12.7 ± 1.8	Unpaired *t*-test	0.595

VD (pixel)	Overall	16.7 ± 1.6	17.0 ± 1.9	Unpaired *t*-test	0.598
	NS	16.8 ± 2.2	16.6 ± 2.4	Unpaired *t*-test	0.780
	NI	15.7 ± 2.5	16.7 ± 3.1	Mann–Whitney *U* test	0.377
	TI	18.1 ± 3.3	18.1 ± 3.8	Unpaired *t*-test	0.973
	TS	16.5 ± 2.9	17.9 ± 2.7	Unpaired *t*-test	0.182
	Macular region	14.9 ± 2.8	16.3 ± 2.3	Unpaired *t*-test	0.131
