# Supplementary material for: Macular Retinal Vessel Oxygen Saturation Elevation in Chinese Central Serous Chorioretinopathy
Source: J Ophthalmol. 2017 Nov 2;2017:5972418. doi: 10.1155/2017/5972418 (PMC5688345; doi:10.1155/2017/5972418)
Supplement: Supplementary file 1 — Table 1. Comparison of oxygen saturation and vessel diameter between CSC affected eyes and healthy young people's normal eyes. Table 2. Comparison of oxygen saturation and vessel diameter between CSC patients' contralateral eyes and healthy young people's normal eyes. [file 5972418.f1.docx]

**Table 1: Comparison of oxygen saturation and vessel diameter between CSC affected eyes and healthy young people’s normal eyes.**

|  | Variables | Affected Eyes | Healthy Eyes | Statistical Method | *P*-value |
| --- | --- | --- | --- | --- | --- |
| AS (%) | Overall | 96.6 ± 7.0 | 93.5 ± 6.8 | Mann-Whitney U test | 0.019* |
|  | NS | 101.2 ± 7.9 | 98.4 ± 9.6 | Mann-Whitney U test | 0.093* |
|  | NI | 98.0 ± 10.2 | 96.2 ± 11.3 | Mann-Whitney U test | 0.274 |
|  | TI | 93.2 ± 10.2 | 89.3 ± 8.8 | Unpaired *t*-test | 0.030* |
|  | TS | 94.5 ± 8.0 | 90.4 ± 9.0 | Mann-Whitney U test | 0.008* |
| VS (%) | Overall | 60.9 ± 6.2 | 60.8 ± 5.8 | Mann-Whitney U test | 0.965 |
|  | NS | 62.4 ± 7.1 | 63.3 ± 7.5 | Unpaired *t*-test | 0.512 |
|  | NI | 61.3 ± 7.3 | 60.2 ± 7.8 | Mann-Whitney U test | 0.357 |
|  | TI | 53.4 ± 8.7 | 58.6 ± 7.8 | Unpaired *t*-test | 0.001* |
|  | TS | 61.1 ± 8.8 | 61.0 ± 7.7 | Mann-Whitney U test | 0.845 |
| AVS (%) | Overall | 35.7 ± 6.5 | 32.6 ± 8.3 | Mann-Whitney U test | 0.024* |
|  | NS | 38.8 ± 9.3 | 35.0 ± 9.9 | Mann-Whitney U test | 0.020* |
|  | NI | 36.7 ± 10.4 | 36.0 ± 12.5 | Mann-Whitney U test | 0.568 |
|  | TI | 39.8 ± 10.7 | 30.7 ± 10.1 | Unpaired *t*-test | 0.000* |
|  | TS | 33.4 ± 10.7 | 29.3 ± 10.5 | Mann-Whitney U test | 0.083 |
| AD (pixel) | Overall | 13.0 ± 1.2 | 13.4 ± 1.3 | Unpaired *t*-test | 0.059 |
|  | NS | 12.8 ± 1.8 | 13.2 ± 1.8 | Unpaired *t*-test | 0.328 |
|  | NI | 12.5 ± 1.8 | 12.9 ± 2.0 | Unpaired *t*-test | 0.278 |
|  | TI | 13.8 ± 2.5 | 14.4 ± 2.3 | Unpaired *t*-test | 0.254 |
|  | TS | 13.4 ± 2.0 | 13.9 ± 2.3 | Mann-Whitney U test | 0.156 |
| VD (pixel) | Overall | 17.3 ± 1.4 | 15.8 ± 1.4 | Unpaired *t*-test | 0.000* |
|  | NS | 17.1 ± 2.1 | 15.7 ± 2.4 | Unpaired *t*-test | 0.002* |
|  | NI | 15.8 ± 2.9 | 15.9 ± 3.0 | Unpaired *t*-test | 0.893 |
|  | TI | 19.9 ± 2.5 | 15.5 ± 3.0 | Unpaired *t*-test | 0.000* |
|  | TS | 18.1 ± 2.7 | 16.8 ± 3.0 | Unpaired *t*-test | 0.030* |

**p* < 0.05.

**Table 2: Comparison of oxygen saturation and vessel diameter between CSC patients’ contralateral eyes and healthy young people’s normal eyes.**

|  | Variables | Contralateral Eyes | Healthy Eyes | Statistical Method | *P*-value |
| --- | --- | --- | --- | --- | --- |
| AS (%) | Overall | 95.4 ± 6.7 | 93.5 ± 6.8 | Mann-Whitney U test | 0.136 |
|  | NS | 98.9 ± 8.1 | 98.4 ± 9.6 | Mann-Whitney U test | 0.700 |
|  | NI | 98.5 ± 11.6 | 96.2 ± 11.3 | Mann-Whitney U test | 0.079 |
|  | TI | 88.7 ± 7.7 | 89.3 ± 8.8 | Unpaired *t*-test | 0.729 |
|  | TS | 93.1 ± 6.2 | 90.4 ± 9.0 | Mann-Whitney U test | 0.044* |
| VS (%) | Overall | 58.8 ± 6.3 | 60.8 ± 5.8 | Mann-Whitney U test | 0.155 |
|  | NS | 61.0 ± 7.6 | 63.3 ± 7.5 | Unpaired *t*-test | 0.110 |
|  | NI | 56.9 ± 6.5 | 60.2 ± 7.8 | Mann-Whitney U test | 0.020* |
|  | TI | 52.8 ± 9.0 | 58.6 ± 7.8 | Unpaired *t*-test | 0.000* |
|  | TS | 61.3 ± 8.6 | 61.0 ± 7.7 | Mann-Whitney U test | 0.531 |
| AVS (%) | Overall | 36.6 ± 6.3 | 32.6 ± 8.3 | Mann-Whitney U test | 0.002* |
|  | NS | 37.9 ± 9.7 | 35.0 ± 9.9 | Mann-Whitney U test | 0.141 |
|  | NI | 41.5 ± 11.2 | 36.0 ± 12.5 | Mann-Whitney U test | 0.002* |
|  | TI | 35.9 ± 9.9 | 30.7 ± 10.1 | Unpaired *t*-test | 0.008* |
|  | TS | 31.7 ± 8.5 | 29.3 ± 10.5 | Mann-Whitney U test | 0.252 |
| AD (pixel) | Overall | 13.0 ± 1.2 | 13.4 ± 1.3 | Unpaired *t*-test | 0.114 |
|  | NS | 13.0 ± 1.8 | 13.2 ± 1.8 | Unpaired *t*-test | 0.629 |
|  | NI | 12.7 ± 1.7 | 12.9 ± 2.0 | Mann-Whitney U test | 0.528 |
|  | TI | 13.8 ± 2.5 | 14.4 ± 2.3 | Unpaired *t*-test | 0.196 |
|  | TS | 14.2 ± 2.0 | 13.9 ± 2.3 | Mann-Whitney U test | 0.304 |
| VD (pixel) | Overall | 16.8 ± 1.7 | 15.8 ± 1.4 | Unpaired *t*-test | 0.001* |
|  | NS | 16.8 ± 2.2 | 15.7 ± 2.4 | Unpaired *t*-test | 0.021* |
|  | NI | 16.1 ± 2.8 | 15.9 ± 3.0 | Mann-Whitney U test | 0.995 |
|  | TI | 18.1 ± 3.4 | 15.5 ± 3.0 | Unpaired *t*-test | 0.000* |
|  | TS | 17.0 ± 2.8 | 16.8 ± 3.0 | Unpaired *t*-test | 0.673 |

**p* < 0.05.

Statistical method: the unpaired *t*-test or rank-sum test (Mann-Whitney U test) was used depending on the distribution of data (Shapiro-Wilk test; α = 0.05) and homogeneity of variance (*F*-test; α = 0.05): Unpaired *t*-test for normal distribution and homogeneous variance, ranks sum test (Mann-Whitney U test) for abnormal distribution or inhomogeneous variance.
